# Physiopathology of necrobiotic xanthogranuloma with monoclonal gammopathy

**DOI:** 10.1111/joim.12195

**Published:** 2014-02-10

**Authors:** R Szalat, J Pirault, J-P Fermand, A Carrié, F Saint-Charles, M Olivier, P Robillard, E Frisdal, E F Villard, P Cathébras, E Bruckert, M John Chapman, P Giral, M Guerin, P Lesnik, W Le Goff

**Affiliations:** 1 Département d'immunologie Clinique, Hôpital Saint LouisParis, France; 2 EA3963, Université Paris 7 Denis Diderot, INSERM, IFR105, Institut Universitaire d'HématologieParis, France; 3 INSERM, UMR_S939, Dyslipidemia, Inflammation and Atherosclerosis in Metabolic DiseasesParis, France; 4 UPMC Univ Paris 06, UMR_S939Paris, France; 5 Institute of Cardiometabolism and Nutrition (ICAN)Paris, France; 6 Assistance-Publique Hôpitaux de Paris, Hôpital Pitié-SalpêtrièreParis, France; 7 Service de médecine interne hôpital NordSaint-Etienne Cedex 2, France

**Keywords:** HDL, histiocytes, immune complex, monocyte, monoclonal immunoglobulin, necrobiotic xanthogranuloma

## Abstract

**Rationale:**

Xanthomatosis associated with monoclonal gammopathy includes hyperlipidaemic xanthoma (HX), normolipidaemic xanthoma (NX) and necrobiotic xanthogranuloma (NXG). All three pathologies are characterized by skin or visceral lesions related to cholesterol accumulation, monoclonal immunoglobulin (MIg) and hypocomplementemia. The pathophysiology underlying NXG remains unknown although the involvement of MIg is suspected.

**Objective:**

To provide further insights into the pathophysiology of NXG, we evaluated the plasma lipid phenotype, mechanisms involved in cellular cholesterol accumulation and role of MIg in an analysis of blood and plasma markers of inflammation in 16 patients with xanthomatosis [NXG (*n* = 8) and NX (*n* = 8)] associated with monoclonal IgG relative to the relevant controls.

**Results:**

The lipid profile of patients with NXG was characterized by a low HDL-C phenotype and an abnormal distribution of HDL particles. Sera from patients with NXG induced cholesterol accumulation in human macrophages. This accumulation was due in part to a significant reduction in the HDL capacity to promote cholesterol efflux from macrophages, which was not found in the case of NX. The MIg of NXG and NX patients was tested positively by ELISA to recognize a large spectrum of lipoproteins. High plasma levels of pro-inflammatory cytokines (TNFα and IL-6), soluble cytokine receptors (sIL-6R, sTNFRI and sTNFRII), adhesion molecules (VCAM-1 and ICAM-1) and chemokines (MCP-1, IL-8 and MIP-1α) were observed in both patients with NXG and NX, revealing a specific xanthoma inflammatory signature which was inversely correlated with plasma levels of anti-inflammatory HDL. However, patients with NXG were distinguished by elevated levels of IL-15 and a marked increase in the rate of intermediate CD14++CD16+ monocytes.

**Conclusion:**

This study revealed that NXG is characterized by impaired macrophage lipid homeostasis associated with a systemic inflammatory profile that may result from the interaction of MIg and lipoproteins.

## Key points

- First characterization of the lipid inflammatory phenotype of patients with xanthomatosis and monoclonal gammopathy in a population of 16 patients.
- NXG is characterized by impaired macrophage lipid homeostasis and is associated with a unique systemic inflammatory profile.

## Introduction

Monoclonal immunoglobulin (MIg) is detected in the serum of approximately 3% of individuals older than 50 years of age 1. MIg may indicate the presence of multiple myeloma or Waldenström macroglobulinemia, or it may not be associated with detectable haematologic disease. In the latter case, MIg is called ‘monoclonal gammopathy of undetermined significance' (MGUS). In some instances, MIg is characterized by antibody activity or particular biochemical processes that may lead to serious organ damage; examples include anti-MAG neuropathy, cold agglutinin disease, acquired C1 inhibitor deficiency or type 2 cryoglobulinaemia 2.

Xanthomas are characterized by the deposition of lipids (mainly cholesterol) in large foam cells primarily in the skin and tendons. Although usually observed in patients displaying dyslipidaemia, xanthomatosis may also occur in cases of MIg 3. In the latter situation, three forms have been identified, namely hyperlipidaemic xanthoma (HX), diffuse plane normolipidaemic xanthoma (NX) and necrobiotic xanthogranuloma (NXG) 4, each of which display distinct clinical and histological characteristics. Xanthoma lesions in NX and HX are yellow maculae or diffuse and plane plaques, whereas lesions are violaceous to red-orange or yellow and form firm papules, nodules or plaques in NXG. NX, HX and NXG lesions are histologically characterized by the presence of foam cells, which have a monocyte–macrophage origin. In NXG, these cells form giant cells called Touton cells, which are associated with necrobiosis, lymphocytes, cholesterol clefts and nodular lymphoid aggregates 3,4. Importantly, the three types of xanthomatosis associated with MIg are associated with complement consumption, suggesting the presence of immune complexes. MIg associated autoantibody activity directed against plasma lipoproteins has been documented in NX and HX 5–10. This activity probably results in the formation of immune complexes and the accumulation of cholesterol derivatives in circulating monocytes and macrophages. However, the pathophysiology of NXG has not yet been described, and the mechanisms underlying its distinct clinical patterns remain unknown, although cholesterol accumulation in monocytes and a role for MIg have been suggested 10,11.

To provide further insight into the physiopathology of NXG, we investigated 16 patients displaying xanthomatosis with monoclonal gammopathy together with relevant control groups. We performed an in-depth assessment of macrophage lipid uptake and lipid export processes to understand the mechanisms of foam-cell formation in addition to an analysis of plasma lipids and lipoprotein profiles, blood leucocyte populations and inflammatory phenotypes.

## Materials and methods

### Patients

**Fig 1:**
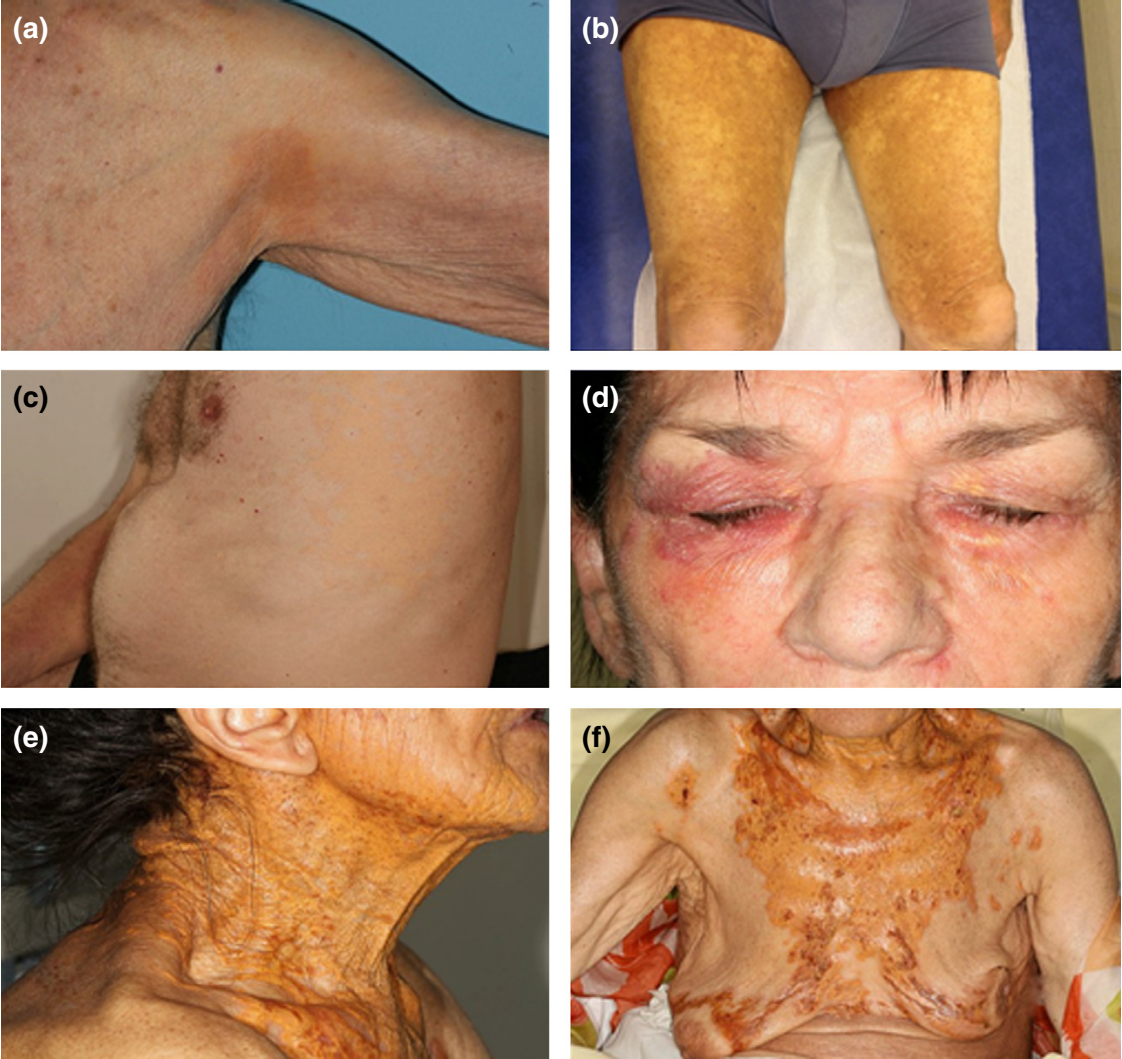
Clinical aspects of patients with NX and NXG. (a) Patients NX1, (b) NX5, (c) NX7, (d) NXG7 and (e–f) NXG8.

Patients with NX (*n* = 8; 3 female patients and five male patients) or NXG (*n* = 8; 6 female patients and two male patients) were diagnosed between 2004 and 2011. At the time of the study, subjects were between 37 and 82 years of age for NX, and 60 and 83 years of age for NXG. The mean ages were 62 ± 5.8 and 68.8 ± 2.4 years for NX and NXG subjects, respectively. Xanthoma lesions, as identified by histological examination, contained foam-cell infiltration or necrobiosis, cholesterol clefts and Touton giant cells in both NX and NXG (Fig.1). All patients had serum MIg, as was detected by electrophoresis and confirmed by immunofixation (IF). The MIg isotype (IgG, 6 κ and 10 λ), hypocomplementaemia and underlying haematological conditions (MGUS, *n* = 9; multiple myeloma, *n* = 6; and lymphoma, *n* = 1) are detailed in Table1. A diagnosis of atherosclerosis was retained if patients had a cardiovascular event in their background, including stroke, arteriopathy or ischaemic cardiopathy, or during follow-up. Arterial Doppler evaluation was also considered when available (*n* = 5) (Table1). No patient suffered from obesity, arterial hypertension or insulin resistance that might suggest a metabolic syndrome 12. Test subjects were compared with three groups of control subjects: (i) sex- and age-matched individuals with negative serum IF and no xanthoma lesions (normal controls, *n* = 8), (ii) sex- and age-matched individuals with detectable serum MIg and no xanthoma lesions (MIg controls, *n* = 5) and (iii) individuals with familial hypercholesterolaemia (FH) who exhibit xanthoma but not MIg and were homozygous (*n* = 3), heterozygous (*n* = 2) or doubly heterozygous (*n* = 2) for point mutations in the *LDLR* and/or *APOB* genes (xanthoma controls, Table S1) 13. Only patients with FH received lipid-lowering drugs and regularly underwent LDL apheresis at intervals of 2–3 weeks. Blood samples from FH patients were collected 2–3 weeks after LDL apheresis and just prior to undergoing a subsequent apheresis. In all cases, blood samples were collected by venipuncture from the antecubital vein into sterile EDTA-containing tubes (final EDTA concentration, 1 mg mL^−1^). Plasma was separated immediately by low-speed centrifugation at 2500 rpm for 20 min at 4 °C and stored at −80 °C until use. The study protocol was approved by the Saint-Louis Hospital Ethic Committee, and the study was conducted in accordance with the ethical principles set forth in the Declaration of Helsinki. Written informed consent was obtained from all patients.

**Table 1:** Clinical characteristics, complement levels and plasma lipid parameters in patients with NX and NXG

PATIENTS	NX1	NX2	NX3	NX4	NX5	NX6	NX7	NX8	NXG1	NXG2	NXG3	NXG4	NXG5	NXG6	NXG7	NXG8
Gender	M	F	F	M	M	M	M	F	M	F	F	F	F	M	F	F
Age (year)	75	37	74	52	42	68	66	82	68	69	65	68	65	60	73	83
Lesion topography	Trunk, palpebral, diffuse	Diffuse xanthodermia	Trunk, peri-palpebral	Trunk, folds	Trunk, folds	Trunk, folds	Diffuse xanthodermia	Trunk	Multiple Skin lesions	Multiple skin lesions	Peri-Orbital	Trunk	Trunk	Retro-Orbital mass+ Peri-Orbital	Peri-orbital	Trunk, Peri-Orbital, multiple skin lesions
Monoclonal isotype	IgG_λ_	IgG_K_	IgG_K_	IgG_λ_	IgG_λ_	IgG_K_	IgG_λ_	IgG_λ_	IgG_λ_	IgG_k_	IgG_k_	IgG_k_	IgG_λ_	IgG_λ_	IgG_λ_	IgG_λ_
Hemopathy	MGUS	Stade 1 MM	MM	MGUS	MGUS	MGUS	MGUS	CLL	MGUS	Stade 1 MM	MGUS	MM	MGUS	MGUS	MM	Stade 1 MM
Treatment	None	Melphalan, Auto-transplant	Bortezomib	None	None	None	None	None	Bortezomib, Thalidomide	Thalidomide	None	Bortezomib Cyclophosphamide	None	None	None	IvIg
CV events	None	None	None	None	None	Severe atherosclerosis/ischaemic cardiopathy/stroke	None	None	Carotid dissection/Ischaemic cardiopathy	None	None	None	None	None	None	None
Complement levels	Low CH50 Low C3 Low C4	Low CH50 Low C4	Low CH50 Low C3 Low C4	Low C4	Low CH50 Low C3 Low C4	Low C4	Low CH50 Low C4	Low CH50 Low C4	Low CH50 Low C4	Low CH50 Low C4	Low CH50 Low C4	Low C4	Low CH50 Low C4 Low C3	Low CH50 Low C3 Low C4	Low CH50	Low CH50 Low C3
Total Cholesterol (mg dL^−1^)	173.4	170.9	257.8	203.1	193.8	182.3	166.9	192.9	157.0	170.2	174.2	170.4	5.5	209.8	188.1	199.6
Free Cholesterol (mg dL^−1^)	54.1	50.1	105.0	59.5	nd	52.2	48.1	52.3	44.7	47.3	53.2	nd	0.00	59.1	50.8	62.6
Triglycerides (mg dL^−1^)	68.5	81.7	470.5	165.0	60.0	58.3	91.8	94.2	160.8	66.4	152.4	143.2	10.4	48.4	125.2	318,5
ApoB (g L^−1^)	0.99	1.00	1.27	1.11	0.77	0.72	0.86	1.04	0.88	0.68	0.76	1.00	0.00	0.89	0.9	1.16
LDL cholesterol (mg dL^−1^)	134.7	134.5	140.7	150.1	113.8	106.7	103.5	135	102.8	90.0	nd	113.8	0.00	134.1	129.1	108.9
ApoA-I (g L^−1^)	0.93	0.94	1.21	0.87	1.30	1.49	1.29	1.08	1.56	1.42	1.56	1.12	0.22	1.21	0.92	1.09
ApoA-II (g L^−1^)	0.15	0.20	0.10	0.14	0.19	0.25	0.33	0.22	0.11	0.16	nd	0.25	0.05	0.14	0.21	0.31
ApoE (mg dL^−1^)	1.82	4.20	17.82	5.26	2.08	2.29	2.31	4.02	1.60	0.78	nd	1.98	0.00	4.18	3.86	5.53
HDL cholesterol (mg dL^−1^)	25.0	20.0	23.0	20.0	68.0	64.0	45.0	39.0	22.0	64.0	nd	28.0	5.0	66.0	34.0	27
CRP (mg L^−1^)	2.6	2.0	10.7	3.1	0.4	22.7	2.3	2.4	30.0	3.00	10.0	1.00	12.5	1.80	3.18	2.23

nd: not determined. MGUS: monoclonal gammopathy of undetermined significance. MM: multiple myeloma. CLL: chronic lymphoid leukaemia.

### Biochemical analysis

The lipid (triglycerides, phospholipids, total cholesterol, free cholesterol and HDL-C) and protein [total protein, apolipoprotein (apo)A-I, apoA-II, apoB and apoE] content of plasma and isolated lipoprotein fractions was quantified with an autoanalyzer (Konelab 20). Reagent kits from Roche Diagnostics (Meylan, France) and Thermo Fisher Scientific (Villebon sur Yvette, France) were used to determine total cholesterol and triglyceride levels, respectively. Unesterified cholesterol and phospholipid levels were measured with commercial reagent kits (Diasys, Condom, France). Cholesteryl ester (CE) mass was calculated as (TC−FC) × 1.67 and thus represented the sum of esterified cholesterol and fatty acid moieties. Bicinchoninic acid assay reagent (Pierce, Perbio Science, Courtaboeuf, France) was utilized for protein quantification. Fasting plasma LDL-C was calculated using the Friedewald formula. HDL-C levels were determined after dextran sulphate–magnesium precipitation of apoB-containing lipoproteins. Plasma apoA-I, apoB and apoA-II concentrations were determined using immunoturbidimetric assays (Thermo Fisher Scientific) reagents and calibrators; Diasys reagents and calibrators.

### Isolation of lipoprotein subfractions

Lipoproteins were isolated from plasma by isopycnic density gradient ultracentrifugation in a Beckman Sw41 Ti rotor at 40 000 rpm for 48 h in a Beckman XL70 at 15 °C as previously described 14. Lipoprotein mass concentration expressed in mg dL^−1^ was calculated as the sum of the concentrations, expressed in mg dL^−1^, of the individual lipid (free cholesterol, esterified cholesterol, phospholipids and triglycerides) and total protein components for each lipoprotein fraction.

### Preparation and culture of human macrophages

Monocytes were isolated on Ficoll gradients (Ficoll-Paque PLUS, GE Healthcare, Villacoublay, France) from the blood of healthy, individual normolipidaemic donors (Etablissement Français du Sang, EFS) and subsequently differentiated into human monocyte-derived macrophages (HMDM) on plastic Primaria plates (BD Biosciences Falcon, Le Pont de Claix, France) over a period of 10 days of culture in RPMI 1640 medium supplemented with 10% heat-inactivated human serum (HS), 2 mmol L^−1^ glutamine, 100 U mL^−1^ penicillin, 100 μg mL^−1^ streptomycin and 20 ng mL^−1^ human macrophage colony-stimulating factor (hM-CSF). A human THP-1 monocytic cell line was obtained from American Type Culture Collection (ATCC) and maintained in culture in 5% CO_2_ at 37 °C in RPMI medium containing 10% heat-inactivated foetal bovine serum, 2 mmol L^−1^ L-glutamine, 80 U mL^−1^ penicillin and 80 μg mL^−1^ streptomycin. THP-1 monocytes were grown in the presence of 50 ng mL^−1^ of phorbol 12-myristate 13-acetate (PMA) for 3 days to induce differentiation into macrophages.

### Determination of cellular cholesterol mass

Human THP-1 macrophages and HMDM were incubated for 48 h in the presence of 10% serum from either patients or control subjects diluted in RPMI media. The mass of esterified and free cholesterol in human macrophages was quantified using an Amplex Red cholesterol assay kit (Molecular Probes, Life Technologies, Saint-Aubin, France) as previously described 15.

### Cell-free cholesterol efflux assays

Human THP-1 macrophages from either patients or control subjects were labelled with 1 μCi mL^−1^ [^3^H]cholesterol for 24 h in the presence of 10% serum diluted in RPMI media or 50 μg mL^−1^ acetylated LDL (acLDL) in RPMI medium containing 2 mmol L^−1^ glutamine, 50 mmol L^−1^ glucose and 0.2% BSA (RGGB media) as indicated. Human macrophages were then equilibrated in RGGB medium for an additional 16 h. Cellular cholesterol efflux in either 2.5% test or control subject serum or 15 μg mL^−1^ of the HDL-PL subfractions was assayed in RGGB media for a 4-h chase period. Finally, culture media was harvested and cleared of cellular debris with a brief centrifugation. Cell-associated radioactivity was determined by extraction in hexan-isopropanol (3 : 2), evaporation of the solvent and liquid scintillation counting (Wallac Trilux 1450 MicroBeta; Perkin Elmer, Courtaboeuf, France). The percentage of cholesterol efflux was calculated as 100 × (medium cpm)/(medium cpm + cell cpm).

### Recognition of lipoprotein-associated antigens with enzyme-linked immunosorbent assay (ELISA)

Lipoprotein subfractions were prepared from normolipidaemic plasmas as described above and used to coat 96-well plates (MaxiSorp ImmunoPlate, Nunc, Thermo Fisher Scientific) overnight at 4 °C (protein concentration, 5 μg mL^−1^). Wells were then washed five times with PBS with 0.05% Tween 20, and blocking was performed for 1 h with PBS containing 1% BSA at room temperature. Diluted plasma samples (1/1000) were incubated for 2 h at room temperature and, after five washing steps, 100 μL of biotinylated mouse anti-human IgG or anti-human Ig kappa light chain or anti-human lambda light chain (1/10 000 dilution, BD Biosciences) was added for 1 h at room temperature. Following incubation, wells were washed five times and incubated with 100 μL Avidin-HRP (1/1000 dilution, eBioscience, Paris, France) for 30 min at RT. After extensive washing (x7), reactions were initiated by adding TMB substrate reagent (eBioscience) for 15 min at room temperature and were stopped by the addition of 50 μL of a 2 N sulphuric acid solution. Signal was quantified by measuring the optical density at 450 nm using a microplate reader (Dynex Technologies, Chantilly, VA, USA).

### Analysis of blood leucocyte subsets by flow cytometry

Fresh blood punctures in EDTA tubes were immediately centrifuged for 20 min at 3000 rpm and 4 °C, and cell pellets were resuspended with an equal volume of cold PBS. A 100- or 300-μL aliquot sample was used for immunostaining of monocytes and lymphocytes or dendritic cells, respectively. Samples were blocked with 200 μL of 1/400 diluted Fc blocking reagent (Miltenyi, Paris, France) and then incubated with corresponding antibodies for 30 min at 4 °C in the dark. If needed, 50 μL of 1/200 diluted streptavidin PE Texas Red (BD Biosciences) was added, and samples were incubated for 15 min at 4 °C in the dark (final dilution, 1/1400). Then, blood red cells were lysed, and leucocytes were fixed with a IO3 fixative/Versalyse mixture (17.5 μL:700 μL for lymphocytes and monocytes, 32.5 μL: 1300 μL for DC) according to the manufacturer's instructions (Beckman Coulter, Roissy CDG, France). The sorting of lymphocyte subsets was based on the different expression patterns of various surface markers. T helper cells expressed CD45+, CD3+, CD4+ and CD8−; naïve T helper lymphocytes expressed CD25− and CD127+; T regulatory cells expressed CD25+ and CD127−; cytotoxic lymphocytes expressed CD45+, CD3+, CD4− and CD8+; B lymphocytes expressed HLA−DR+ and CD 19+. Monocyte subsets were classified as classical (CD14++/CD16−), intermediary (CD14++/CD16+) and nonclassical (CD14+/CD16++) monocytes. DC subsets were classified as plasmacytoid (CD11c−, CD123+ and BDCA2+), myeloid 1 (CD11c+, BDCA1+ and BDCA3−) and myeloid 2 (CD11c+, BDCA1− and BDCA3+). Samples were acquired on an LSR II FORTESSA SORP (BD Biosciences) and analysed using facsdiva software (BD Biosciences). Absolute quantification of leucocytes was assessed using the TRUCOUNT method (BD Biosciences).

### Analysis of circulating biomolecules

Adhesion molecules and inflammatory cytokines in plasma from patients and control subjects were quantified using a semi-automated Biochip Array Technology analyzer (Evidence Investigator, Randox, Crumlin, UK) as previously described 16.

### RNA extraction, reverse transcription and quantitative PCR

Circulating CD14-positive monocytes were isolated from fresh blood samples by automated, magnetic separation using a KingFisher instrument (Thermo Fisher Scientific) and magnetic beads coated with anti-CD14 monoclonal antibody (Dynabeads CD14, Life Technologies). Total RNA was extracted using TRIzol reagent and purified using a NucleoSpin RNA II kit (Macherey-Nagel, Hoerdt, France) according to the manufacturer's instructions. Reverse transcription of RNA and real-time quantitative PCR were performed as previously described 17. mRNA amounts for genes of interest were normalized to six different housekeeping genes (human δ-aminolevulinate synthase, human α-tubulin, human hypoxanthine phosphoribosyltransferase 1, human heat-shock protein 90 kDa alpha, human 18S rRNA and human non-POU domain containing octamer binding). Data were expressed as the fold change in mRNA expression relative to the control values.

### Statistical analyses

Data are presented as the mean ± SEM. Experiments were performed in triplicate. Comparisons of two groups were performed with a two-tailed Student's *t-test* or Mann–Whitney U-test according to the values distribution, and comparisons of three or more groups were performed by anova with a Dunnett post-test. All statistical analyses were performed using the Prism module in the graphpad software package (San Diego, CA, USA).

## Results

### Patients with either NX or NXG display low plasma HDL cholesterol

Both patients with NX and NXG had total plasma cholesterol, apoB and LDL cholesterol levels in the normal range (Table2). Notably, patient with NX3 displayed elevated plasma triglyceride levels, and patients with NXG5 were characterized by severe hypolipidaemia (Table1). More strikingly, patients with NX and NXG displayed low HDL-C levels (<40 mg dL^−1^) relative to the normal range (5 NX/8 and 5 NXG/7). Consistent with the low HDL-C phenotype, plasma apoA-I and apoA-II levels were reduced in both patients with NX (1.12 ± 0.08 g L^−1^, *P* = 0.008 and 0.20 ± 0.07 g L^−1^, *P* = 0.0005, respectively) and NXG (1.14 ± 0.15 g L^−1^ and 0.18 ± 0.03 g L^−1^, *P* = 0.0006, respectively) relative to control subjects (1.47 ± 0.07 and 0.35 ± 0.02 g L^−1^, respectively) (Table2). Such a low HDL phenotype may underlie altered reverse cholesterol transport (RCT) from skin macrophages 18,19 and therefore was evaluated next, because impaired lipid homeostasis in tissue macrophages is frequently observed in pathologies associated with cellular lipid deposits 20.

**Table 2:** Plasma lipid parameters and CRP values in patients with NX and NXG

	NX (*n* = 8)	NXG (*n* = 8)	Controls (*n* = 8)	P NX versus Ctrl	P NXG versus Ctrl	P NX versus NXG
Gender	5M/3F	2M/6F	5M/3F			
Age (year)	62.0 ± 5.8	68.8 ± 2.4	61.3 ± 3.9			
Total cholesterol (mg dL^−1^) (160–260)	191.5 ± 10.7	159.4 ± 22.8	192.7 ± 12.4	0.938	0.218	0.223
Free cholesterol (mg dL^−1^)	59.6 ± 7.75	53.0 ± 2.80	56.2 ± 4.28	0.713	0.549	0.448
Triglycerides (mg dL^−1^) (45–190)	135.7 ± 49.3	128.2 ± 33.3	88.3 ± 7.2	0.373	0.281	0.901
Apolipoprotein B (g L^−1^) (0.60–1.30)	0.96 ± 0.06	0.90 ± 0.06	0.92 ± 0.07	0.661	0.808	0.470
LDL cholesterol (mg dL^−1^) (70–140)	125.6 ± 5.99	113.1 ± 6.72	124.1 ± 12.6	0.915	0.499	0.192
Apolipoprotein A-I (g L^−1^) (1.20–1.70)	1.12 ± 0.08	1.14 ± 0.15	1.47 ± 0.07	**0.008**	0.073	0.939
Apolipoprotein A-II (g L^−1^)	0.20 ± 0.07	0.18 ± 0.03	0.35 ± 0.02	**0.0005**	**0.0006**	0.586
Apolipoprotein E (mg dL^−1^)	5.01 ± 1.88	2.99 ± 074	4.93 ± 0.64	0.969	0.070	0.344
HDL cholesterol (mg dL^−1^) (40–65)	38.4 ± 15.6	35.1 ± 17.1	52.9 ± 4.8	0.101	0.112	0.764
CRP (mg L^−1^) (<5)	5.77 ± 2.65	7.96 ± 3.48	0.73 ± 0.21	**0.005**	**0.001**	0.721

The range of reference values appears within brackets.

Bold values indicate P < 0.05.

### Impaired capacity of sera from patients with NXG to promote free cholesterol efflux from human macrophages

**Fig 2:**
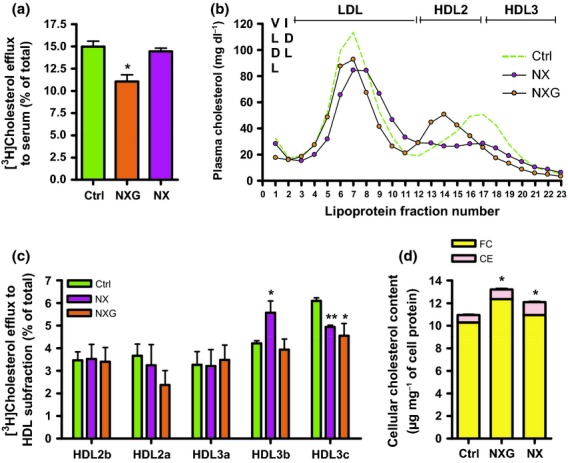
Impaired macrophage lipid homeostasis in patients with NXG and NX. (a) Free [^3^H]cholesterol efflux from human THP-1 macrophages in response to control (n = 4), NXG (n = 6) or NX (n = 8) sera. *P < 0.05 relative to control sera. (b) Plasma lipoprotein profile from control (n = 3), NX (n = 4) and NXG (n = 3) subjects as determined by isopycnic density gradient ultracentrifugation. (c) Analysis of HDL subfraction capacity isolated from control (n = 3), NX (n = 4) and NXG (n = 3) plasma to promote free [^3^H]cholesterol efflux from human THP-1 macrophages. **P < 0.005 relative to the control HDL subfraction. (d) Cellular cholesterol content in human THP-1 macrophages incubated for 48 h with serum from normolipidaemic (control, n = 3), NX (n = 8) or NXG subjects (n = 5). *P < 0.05 versus control sera.

Low plasma HDL cholesterol levels indirectly suggest that accumulation of lipids in the skin of patients with NX and NXG might be associated with a lower capacity of serum to promote RCT. Indeed, as is shown in Fig.2a, sera from patients with NXG exhibited a markedly reduced capacity to promote free cholesterol efflux from human THP-1 macrophages (−33%, *P* < 0.05) relative to control sera. In contrast, THP-1 macrophages displayed a normal cholesterol efflux capacity when incubated with sera from patients with NX. Analysis of lipoprotein profiles (Fig.2b) revealed that, although the distribution of apoB-containing lipoproteins (i.e. VLDL and LDL) appeared similar in patients and controls subjects, the profile of HDL particles (i.e. HDL2 and HDL3, the major physiological cholesterol acceptors from cells) was profoundly altered in both patients with NX and NXG. Indeed, plasma HDL2 and HDL3 levels were markedly reduced in NX subjects, whereas, in patients with NXG, a reduction in plasma HDL3 levels was concomitantly observed with an increase in large HDL2 particles (Fig.2b). Finally, following separation of HDL particles into five individual subfractions by isopycnic density gradient ultracentrifugation, we clearly observed that the capacity of the small HDL3c from both patients with NXG and NX to promote free cholesterol efflux from human THP-1 macrophages was reduced (−25%, *P* < 0.005 and −19%, *P* < 0.05, respectively) relative to HDL3c from control individuals (Fig.2c). This effect might be compensated for in patients with NX by the increased efflux capacity of HDL3b particles.

### Sera from patients with NX and NXG contribute to cholesterol accumulation in human macrophages

Conversely, an alternative mechanism for the specific accumulation of lipids in macrophages was represented by the capacity of serum from NX and NXG individuals to promote the loading of cholesterol in human macrophages. Interestingly, as shown in Fig.2d, the incubation of human THP-1 macrophages with serum from patients with NXG or NX was accompanied by a significant increase in cellular cholesterol content in those cells (+20% and +10%, *P* < 0.05, respectively) relative to sera from normolipidaemic control individuals. Similar observations were made in primary human monocyte-derived macrophages, although this effect was not observed with serum from individuals with monoclonal gammopathy in the absence of xanthoma (Figure S1).

### Interactions between monoclonal Immunoglobulin and lipoproteins

To characterize the antigen specificity of MIg, an ELISA was performed with patients' sera using various purified lipoprotein subfractions as substrates. With this approach, we addressed the hypothesis that the elevated cholesterol deposition in macrophages observed in NXG might result from increased recognition of a specific antigen on lipoproteins by MIg. When sera were diluted at 1:1000 to obtain an MIg serum concentration of 1–10 mg L^−1^, sera from patients with NXG and NX were able to recognize a wide spectrum of lipoprotein subfractions relative to both normal and MIg control subjects. The degree of recognition varied from 2- to 476-fold between patients (Table3). Importantly, this result was confirmed by identifying the corresponding isotype (kappa or lambda) of each patient. Notably, the highest affinity was typically observed with LDL-3 (3/6 for NXG and 4/8 for NX) and, to a lesser extent, oxidized LDL (oxLDL, 2/6 for NXG and 1/8 for NX). These analyses demonstrated that neither patients with NXG nor patients with NX were associated with a specific antigen recognition pattern.

**Table 3:** Recognition of lipoprotein-associated antigens by ELISA in patients with NXG and NX

Patients (MIg Isotype)	IgG	Ranking for recognition of lipoprotein-associated antigens (fold Induction versus control)							
		1th	2nd	3rd	4th	5th	6th	7th	8th
NXG2 (κ)	λ	LDL-5 (8.0)	LDL-3 (5.9)	LDL-2 (5.0)	IDL (4.0)	LDL-4 (2.4)			
	κ	LDL-3 (6.6)	LDL-4 (2.5)						
NXG4 (κ)	λ	LDL-3 (29.3)							
	κ	LDL-3 (16.1)	IDL (3.6)	LDL-1 (3.0)	VLDL (2.3)				
NXG5 (λ)	λ	LDL-3 (475.7)	oxLDL (356.0)	LDL-2 (341.7)	VLDL (120.6)	IDL (81.2)	LDL-5 (74.3)	LDL-4 (27.9)	LDL-1 (13.2)
	κ								
NXG6 (λ)	λ	oxLDL (48.0)	LDL-5 (5.3)	LDL-3 (2.1)					
	κ	IDL (4.0)	LDL-3 (2.5)						
NXG7 (λ)	λ	LDL-4 (2.2)							
	κ								
NXG8 (λ)	λ	oxLDL (6.8)	IDL (3.8)	VLDL (6.1)	LDL-2 (4.2)				
	κ	IDL (18.5)	LDL-5 (9.0)	LDL-1 (6.3)	VLDL (4.3)				
NX1 (λ)	λ	LDL-3 (129.1)	LDL-2 (96.4)	VLDL (64.8)	oxLDL (63)	LDL-5 (46.1)	IDL (38.7)	LDL-4 (8.0)	LDL-1 (4.3)
	κ								
NX2 (κ)	λ	LDL-2 (6.5)	LDL-5 (3.4)						
	κ	oxLDL (13.0)	LDL-3 (12.1)	VLDL (10.5)	IDL (9.9)	LDL-2 (8.2)	LDL-5 (5.8)	LDL-1 (4.7)	LDL-4 (4.0)
NX3 (κ)	λ	LDL-3 (36.8)	LDL-2 (17.5)	LDL-4 (2.3)					
	κ	LDL-3 (3.1)	LDL-2 (3.1)	LDL-4 (2.7)	VLDL (2.5)	IDL (2.4)	LDL-5 (2.0)		
NX4 (λ)	λ	LDL-3 (27.2)	oxLDL (10.5)	LDL-5 (9.1)	VLDL (3.3)	IDL (2.1)	LDL-4 (2.1)		
	κ	IDL (2.8)							
NX5 (λ)	λ	LDL-3 (31.5)	LDL-2 (17.9)	VLDL (10.5)	IDL (6.2)	LDL-5 (5.7)	LDL-4 (3.5)	oxLDL (2.7)	
	κ								
NX6 (κ)	λ	oxLDL (5.1)	LDL-5 (4.6)						
	κ	IDL (49.3)	VLDL (4.6)	LDL-2 (3.4)					
NX7 (λ)	λ	LDL-5 (10.7)	oxLDL (6.4)	VLDL (5.3)	IDL (3.3)	LDL-4 (2.4)			
	κ	LDL-3 (14.4)							
NX8 (λ)	λ	LDL-2 (7.6)	LDL-5 (4.6)	oxLDL (3.5)	IDL (2.0)				
	κ	LDL-5 (3.0)	VLDL (2.0)						

Results are expressed as the fold increase relative to control plasma (*n* = 8).

### Subpopulations of circulating mononuclear cells in patients with NX and NXG

**Fig 3:**
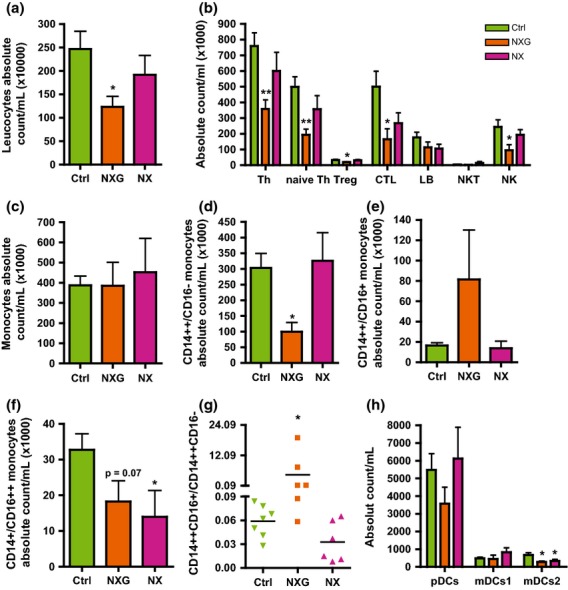
Phenotyping blood leucocytes in patients with NXG and NX. Absolute counting of (a) CD45-positive leucocytes, (b) T lymphocyte subsets (T helper, Th: CD3+ CD4+; naive Th: CD3+ CD4+ CD25+ CD127+; T regulatory cells, Treg: CD3+ CD4+ CD25^hi^ CD127−; cytotoxic T lymphocytes, CTL: CD3+ CD8+; B lymphocytes, LB: CD3− CD19+; NKT: CD3+ CD16+ and natural killer cells, NK: CD3− CD56+), (c) total monocytes, (d) classical monocytes CD14++CD16−, (e) intermediate monocytes CD14++CD16+, (f) nonclassical monocytes CD14+/CD16++, (g) ratio of intermediate monocytes CD14++CD16+/classical monocytes CD14++CD16− and (h) dendritic cell subsets (plasmacytoid DC, pDC: CD11c− CD123+ BDCA2+; myeloid DC1, mDC1: CD11c+ BDCA1+ BDCA3−; and myeloid DC2, mDC2: CD11c+ BDCA1− BDCA3+) by flow cytometry (LSR II Fortessa Sorp, BD Biosciences) in control (no MIg or xanthoma) (n = 7, green), NXG (n = 6, orange) and NX (n = 6, violet) subjects. *P < 0.05 and **P < 0.005 versus control individuals.

Circulating CD45-positive mononuclear leucocytes were analysed by flow cytometry (Fig.3). The number of total circulating mononuclear leucocytes was not different in patients with NX relative to controls, whereas it was reduced by 57% (*P* < 0.05) in patients with NXG (Fig.3a). Neutrophil counts were similar in all groups (Figure S2). Absolute counts of T helper lymphocytes (Th), naive T helpers (naive Th), regulatory T cells (Treg), cytotoxic T lymphocytes (CTL) and natural killer cells (NK) were reduced in patients with NXG [by −53%, −61% (*P* < 0.005), −42%, 77% and −61% (*P* < 0.05), respectively], whereas the number of lymphocytes (LB) and natural killer T cells (NKT) was not altered (Fig.3b) relative to control individuals. The absolute count of monocytes was nearly the same for all patients (Fig.3c), but the absolute count of classical CD14++/CD16− monocytes was markedly reduced for NXG (by −67%, *P* < 0.005) but not NX (Fig.3d). The number of intermediate CD14++/CD16+ monocytes was not significantly different for NXG and NX (Fig.3e), whereas the number of nonclassical CD14+/CD16++ monocytes was reduced (by −44%, *P* = 0.07 for NXG and −57%, *P* < 0.05 for NX) (Fig.3f). More strikingly, the intermediate/classical monocyte ratio was 76-fold higher for NXG than for the controls, whilst no effect was observed for NX (Fig.3g). Finally, analysis of dendritic cells subsets revealed a reduction in the Th2-promoting mDC2 subpopulation in both NX and NXG (−59% and −51%, *P* < 0.05, respectively), whilst absolute counts of the pDC and Th1-promoting mDC1 subsets were not significantly different from control subjects (Fig.3h).

Our findings revealed that patients with NXG are characterized by a leucopenia, which was primarily caused by a reduced number of circulating lymphocytes (Th, naive Th, CTL and NK) and a marked increase in the intermediate/classical monocyte ratio.

### Inflammation markers in the plasma of patients with NX and NXG

**Fig 4:**
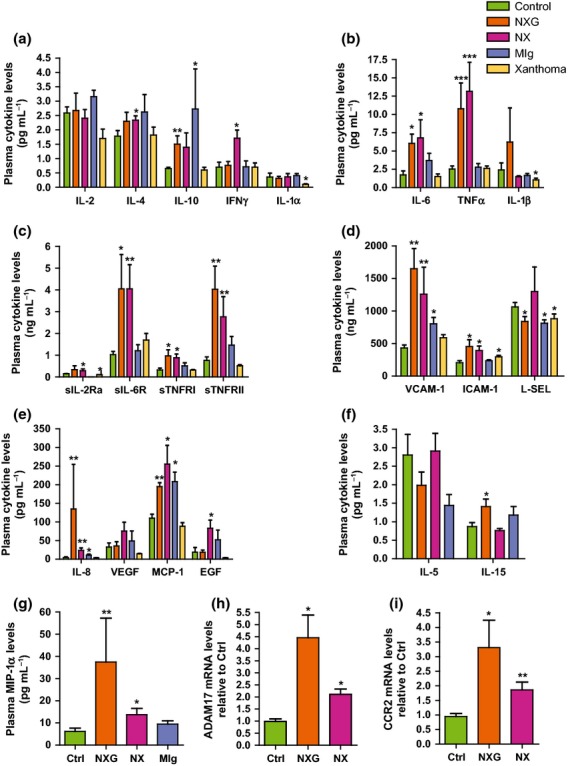
Patients with NX and NXG exhibit a unique inflammatory pattern. Circulating levels of cytokines (a–b, e, f and g), soluble receptors (c) and adhesion molecules (d) were quantified in plasma from normolipidaemic control (no MIg or xanthoma) (n = 8, open bars), NXG (n = 8, dark bars), NX (n = 8, light grey bars), MIg controls (no MIg or xanthoma) (dark grey bars) and xanthoma controls (FH patients without MIg and with xanthoma) (n = 7, hatched bars) individuals as determined on a semi-automated biochip array analyzer (Randox). *P < 0.05, **P < 0.005 and ***P <0.0005 versus control plasma. Amounts of ADAM17 (h) or CCR2 (i) mRNA in freshly isolated CD14-positive monocytes from control (n = 9), NX (n = 6) and NXG (n = 4) subjects. *P < 0.05 relative to the control.

Serum CRP levels were slightly elevated in patients with NXG and NX (7.96 ± 3.48 and 5.77 ± 2.65 mg L^−1^, respectively) (Table2). Circulating inflammatory cytokines and adhesion molecules were quantified using biochip arrays (Fig.4). Results were compared with data obtained with plasma from both normolipidaemic controls with or without MIg and patients with FH who had xanthoma and high plasma cholesterol levels but no detectable monoclonal gammopathy. In patients with NX and NXG, a marked increase in the plasma levels of pro-inflammatory cytokines (TNFα and IL-6) (Figs4b), soluble cytokine receptors (sIL-6R, sTNFRI and sTNFRII) (Fig.4c) and adhesion molecules (VCAM-1 and ICAM-1) (Fig.4d) was observed relative to all control groups. Levels of the plasma chemokines MCP-1, MIP-1α and IL-8 were also elevated in patients with NX and NXG. A similar increase in MCP-1 and IL-8 levels was also observed in normolipidaemic controls with MIg (Fig.4e). Although patients with NX displayed an overlapping pro-inflammatory pattern with patients with NXG, some differences were detected. For example, the NXG pattern was distinguished by an increase in plasma IL-15 levels (+62%, *P* < 0.05) (Fig.4f), whilst the NX pattern was characterized by an elevation of both plasma IFNγ and EGF levels (2.4- and 4.4-fold, respectively, *P* < 0.05) (Figs4a, e). Of note, plasma IFNγ levels were positively correlated with EGF levels in patients with NX and NXG (*r* = −0.412, *P* < 0.05).

Such elevated quantities of circulating TNFα, sIL-6R, sTNFRI, sTNFRII, VCAM-1 and ICAM-1 suggest an increase in the activity of A disintegrin and metalloproteinase (ADAM) 17 and/or 10, each of which are membrane-bound enzymes participating in the shedding of various surface proteins 21. This was confirmed by analysing freshly isolated CD14-positive monocytes from patients with NX and NXG for ADAM17 mRNA expression. This analysis revealed elevated ADAM17 mRNA levels in NX and NXG monocytes relative to control cells (2.1- and 4.5-fold, respectively, *P* < 0.05) (Fig.4h). By contrast, ADAM10 mRNA levels were not altered in patients when compared to controls (data not shown).

High-density lipoproteins have potent anti-inflammatory properties and have been observed to repress endothelial expression of VCAM-1, ICAM-1 22 and chemokine receptors, such as CCR2 23 in circulating monocytes. Consistent with the low HDL-C levels observed in patients with NX and NXG, CCR2 mRNA levels were significantly elevated in circulating CD14-positive monocytes from patients with NX and NXG relative to control individuals (3.5-fold, *P* < 0.05 and 2-fold, *P* < 0.005, respectively) (Fig.4i). Moreover, we observed that plasma HDL and/or apoA-I and apoA-II levels were negatively correlated with the mRNA levels of adhesion molecules (VCAM-1 and ICAM-1), chemokines (MCP-1, MIP-1α, IL-8) and chemokine receptors (CCR2) in CD14-positive monocytes and soluble receptors (sIL-6R, sTNFRI and sTNFRII) in the presently studied patient cohort (Table4).

**Table 4:** Correlation of the HDL phenotype with macrophage lipid homeostasis and the inflammatory profile in the study population

		HDL phenotype		
		HDL-C	ApoA-I	ApoA-II
Macrophage lipid Homeostasis	Free cholesterol efflux	0.61^*^^*^	0.50^*^	0.53^*^
	Cholesterol loading	**−**0.19	−0.12	−0.36
Monocyte recruitment To skin	CCR2 (CD14+ cells)	−0.64^*^	−0.70^*^	−0.38
	MCP-1	−0.48^*^	−0.46^*^	−0.53^*^^*^
	MIP-1α	−0.54^*^	−0.74^*^^*^^*^	−0.54^*^^*^
	IL-8	−0.46^*^	−0.66^*^^*^^*^	−0.44^*^
	VCAM-1	−0.21	0.51^*^	−0.52^*^
	ICAM-1	−0.48^*^	−0.75^*^^*^^*^	−0.59^*^^*^
Soluble receptors	sIL-6R	−0.68^*^^*^^*^	−0.70^*^^*^^*^	−0.71^*^^*^^*^
	sTNFRI	−0.44^*^	−0.64^*^^*^	−0.19
	sTNFRII	−0.27	−0.46^*^	−0.09
NXG	IL-15	−0.24	−0.02	0.67^*^
NX	IFNγ	−0.27	−0.25	−0.46^*^
NX	EGF	−0.35	−0.29	−0.24
Inflammatory cytokines	IL-6	−0.32	−0.37	−0.41^*^
	IL-10	−0.39	−0.41^*^	−0.16
	TNFα	−0.45^*^	−0.66^*^^*^^*^	−0.47^*^

^*^P < 0.05, ^*^^*^P < 0.05 and ^*^^*^^*^P < 0.0005.Control: n = 8, NXG: n = 8, NX: n = 8 and control with MIg: n = 5. Pearson correlation (r).Bold values indicate that correlation values are significant.

## Discussion

The pathophysiology of NXG is not currently well understood. However, a role for MIg is strongly suggested by an almost constant complement consumption that may result from immune complex formation, as is the case for NX or HX, where MIg autoantibody activity has already been documented 5–9. Importantly, NXG is distinguished from NX and HX by different clinical and histological properties that may result from different mechanisms. NXG lesions are characterized by plaques or nodules, and skin infiltration is characterized by the presence of foam macrophage-derived cells, giant cells (Touton cells), necrobiosis and, often, lymphoid aggregates 4. Alternatively, lesions in NX and HX are usually simple and characterized histologically by just foam-cell infiltration.

In our study, NX and NXG exhibited a similar plasma lipid profile that was typically characterized by a low HDL pattern (<40 mg dL^−1^). Probing the effects on monocytes and macrophages of serum from patients with cholesterol homeostasis indicated that sera from patients with NX and NXG promoted cholesterol accumulation in human macrophages, either in a cell line or in normal primary human monocyte-derived macrophages. Interestingly, free cholesterol efflux from macrophages in response to NXG sera was reduced. This effect very likely resulted from a reduction in both the quantity (HDL-C levels were <40 mg dL^−1^) and cholesterol efflux capacity of HDL particles. HDL3c, the most efficient HDL subfraction for the promotion of cholesterol efflux from human macrophages, was particularly important 24. In the case of NX, cholesterol efflux was not reduced, despite a low HDL profile caused by the increased cholesterol efflux capacity of some cholesterol acceptors from the HDL family, particularly HDL3b, which compensates for the reduced efflux capacity of HDL3c.

A distinct antibody activity for MIg in NXG and NX may explain pathophysiological differences observed in those patients. However, ELISA analyses of the interaction between patients' sera and the apoB-containing lipoproteins that are responsible for cellular cholesterol accumulation revealed interactions in all NX and NXG cases without specificity for a unique lipoprotein subclass. The dilution of sera (1 : 1000) together with the kappa/lambda specificity of antigen recognition strongly promoted specific interactions between MIg and lipoproteins. This interaction is likely to produce immune complexes that enter into macrophages through phagocytosis, possibly via scavenger or Fcγ receptors, resulting in cholesterol accumulation.

Whatever the mechanism, cholesterol accumulation in macrophages is a common feature of NX and NXG and cannot explain *per se* the different clinical pattern that characterizes the two conditions. We hypothesized that the distinct phenotype differences between NX and NXG might be due to a different inflammatory profile. By definition, the patients with NX and NXG in our study population had monoclonal gammopathy in the form of MGUS in nine cases, smouldering or overt multiple myeloma (MM) in three cases each and CLL in one case. To rule out a role for underlying monoclonal gammopathy in the inflammatory profile, we compared patients with NX and NXG with a control population with monoclonal gammopathy but no xanthomatosis (MIg controls) and a population with xanthomatosis but no gammopathy (xanthoma controls, i.e. FH patients). In contrast to our patients, cytokine and chemokine levels were close to normal in MIg and xanthoma controls.

Interestingly, we observed that both forms of normolipidaemic xanthomatosis were associated with a common inflammatory pattern characterized by increased CRP and plasma levels of classical pro-inflammatory cytokines (IL-6 and TNFα). In addition, plasma levels of adhesion molecules (VCAM-1 and ICAM-1), chemokines (MCP-1, MIP-1α and IL-8) and soluble cytokine receptors (sTNFRI, sTNFRII and sIL-6R) were also elevated, resulting in a unique inflammatory pattern. This ‘xanthoma inflammatory profile’ might have resulted, at least in part, from increased expression and subsequent shedding of the membrane protein ADAM17. Indeed, TNFα, IL-6R, TNFRI, TNFRII, ICAM-1 and VCAM-1 are substrates of ADAM17, and *ADAM17* expression was elevated in circulating monocytes from patients with NX and NXG relative to controls, whereas the expression of *ADAM10*, which shares common substrates with ADAM17 25, was normal. We observed only slight differences between NX and NXG inflammatory markers, the most important of which was an increase in IFNγ and EGF levels in the case of NX and a higher IL-15 level in the case of patients with NXG. IL-15 may promote monocyte activation and participate in giant- or Touton-cell formation, a typical feature of NXG, because IL-15 is a cytokine known to enhance phagocytic activity 26 and inflammation of monocytes and macrophages by increasing the secretion of pro-inflammatory cytokines and chemokines 27.

Importantly, in agreement with the anti-inflammatory properties of HDL particles and their role in atherosclerosis 22,23, monocyte recruitment markers and soluble receptors were inversely correlated with plasma HDL-C and apoA-I in patients with NX and NXG. These observations revealed a particular chemokine and cytokine environment that was intimately linked to the HDL phenotype observed in those patients. However, cardiovascular events were recorded in only two patients of the present cohort and have not been reported often in the literature 4. Therefore, patients with NXG and NX appear to have relatively little atherosclerosis. Why lipid-loaded macrophage cells preferentially reside in cutaneous sites without a significant vascular tropism remain to be determined.

Finally, in NXG but not NX, we observed an increased number of intermediate CD14++CD16+ monocytes and a marked reduction in that of classical CD14++CD16− monocytes, leading to a massive elevation in the proportion of intermediate to classical monocytes. Such an observation in NXG is particularly interesting because the specific intermediate CD14++CD16+ monocyte subset has been recently reported to be deleterious in pathological conditions 28–32. Indeed, in addition to the high proinflammatory phenotype of this monocyte subset, intermediate CD14++CD16+ monocytes selectively express the C-C chemokine receptor type 5 (CCR5) 33,34, which binds chemokine ligands such as MIP-1α. Then, the increase in this specific CCR5-expressing monocyte subset combined with the high concentration of blood MIP-1α in NXG may favour the recruitment of circulating monocytes in skin lesions of patients with NXG. Finally, gene ontology enrichment analysis linked intermediate CD14++CD16+ monocytes to antigen processing and presentation, inflammation and monocyte activation 33. These properties taken together may also contribute to the clinical and histological differences between both NXG and NX and, in particular, the more pronounced cutaneous inflammation that characterized NXG subjects.

Further study is required to better understand the link between elevated levels of circulating IL-15, the intermediate CD14++CD16+ monocyte subset, MIg, impaired macrophage cholesterol homeostasis and the inflammatory phenotype in physiopathology of NXG.

**Fig 5:**
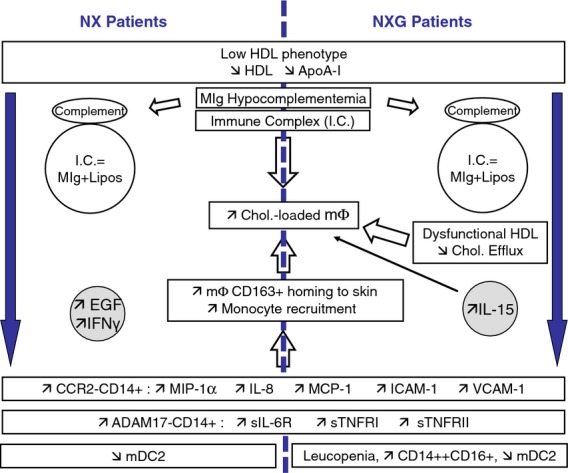
Proposed molecular and cellular mechanisms for the appearance of skin lesions in patients with NX and NXG. Low levels of anti-inflammatory HDL-C (apoA-I) were associated with increased expression of chemokine receptor CCR2 in circulating monocytes and elevated levels of chemokines (MCP-1, IL-8, MIP-1α) and adhesion molecules (VCAM-1, ICAM-1), which might favour monocyte recruitment and homing to skin in patients with NX and NXG. This effect might be exacerbated in the case of NXG due to the presence of a high proportion of intermediate CD14++CD16+ monocytes. Moreover, increased levels of soluble receptors (sIL-6R, sTNFRI and TNFRII), which were also inversely correlated with plasma HDL-C (apoA-I) levels, contributed to the ‘xanthoma inflammatory profile’ observed in patients with NX and NXG. Although serum from both NX and NXG promoted cholesterol accumulation in macrophages, the appearance of foam cells in NXG likely also resulted from the impaired capacity of HDL particles (HDL3c) to promote macrophage cholesterol efflux. In addition, the presence of immune complexes (IgG anti-lipoproteins) that might enter into macrophage through phagocytosis via scavenger or Fcγ receptors may also contribute to foam-cell formations in both NXG and NX. Finally, the differential levels of EGF, IL-15 and IFNγ observed in NX and NXG subjects could provide some preliminary clues to help explain the mechanisms underlying the distinct clinical patterns that characterize NX and NXG.

In conclusion, our present findings are integrated schematically in Fig.5. Herein, we report that NXG is characterized by an abnormal HDL phenotype associated with a unique inflammatory pattern, the preponderance of a specific monocyte subset and an impaired macrophage cholesterol homeostasis, probably due to interactions between MIg and lipoproteins. Although NXG is a relatively rare condition, our results contribute to the understanding of the cellular mechanisms involved in lipid accumulation.

## Abbreviations

ADAM: A disintegrin and metalloproteinase
apo: apolipoprotein
CCR2: chemokine (C-C motif) receptor 2
CE: cholesteryl esters
DC: dendritic cells
EGF: epidermal growth factor
FC: free cholesterol
FH: familial hypercholesterolaemia
HDL: high-density lipoprotein
HMDM: human monocyte-derived macrophages
ICAM-1: intercellular adhesion molecule-1
IFNγ: interferon-gamma
IL: interleukin
LDL: low-density lipoprotein
MCP-1: monocyte chemotactic protein-1
MIg: monoclonal immunoglobulin
MIP-1α: macrophage inflammatory protein-1α
NX: normolipidaemic xanthoma
NXG: necrobiotic xanthogranuloma
TNFα: tumour necrosis factor-alpha
VCAM-1: vascular cell adhesion molecule-1

## Supporting Information

Table S1 Plasma lipid parameters and CRP values in Familial Hypercholesterolaemia patients (Xanthoma controls). **Figure S1** Impact of sera from NXG and NX patients on cholesterol content in human monocyte-derived macrophages. **Figure S2** Blood neutrophils in NXG and NX patients.
