# Supplementary material for: Physiopathology of necrobiotic xanthogranuloma with monoclonal gammopathy
Source: J Intern Med. 2014 Feb 10;276(3):269–84. doi: 10.1111/joim.12195 (PMC4279948; doi:10.1111/joim.12195)
Supplement: Table S1 — Plasma lipid parameters and CRP values in Familial Hypercholesterolaemia patients (Xanthoma controls). Figure S1 Impact of sera from NXG and NX patients on cholesterol content in human monocyte-derived macrophages. Figure S2 Blood neutrophils in NXG and NX patients. [file joim0276-0269-SD1.docx]

|  |  |
| --- | --- |
|  |  |
|  | **FH**  **(n=7)** |
|  |  |
| **Gender** | 3M / 4F |
| **Age (year)** | 38.8 ± 5.9 |
| **Total Cholesterol (mg/dL)**  (160-260) | 295.9 ± 23.5 |
| **Free Cholesterol (mg/dL)** | 101.0 ± 7.0 |
| **Triglycerides (mg/dL)**  (45-190) | 101.0 ± 16.3 |
| **Apolipoprotein B (g/L)**  (0.60-1.30) | 1.56 ± 0.15 |
| **LDL-Cholesterol (mg/dL)**  (70-140) | 234.9 ± 23.7 |
| **Apolipoprotein A-I (g/L)**  (1.20-1.70) | 1.57 ± 0.09 |
| **Apolipoprotein E (mg/dL)** | 3.80 ± 0.30 |
| **HDL-Cholesterol (mg/dL)**  (40-65) | 40.7 ± 4.20 |
| **CRP (mg/L)**  (<5) | 1.40 ± 0.90 |

**Table S1: Plasma lipid parameters and CRP values in Familial**

**Hypercholesterolemia patients (Xanthoma controls). Range of**

**Reference values between brackets.**
